# Polycythemia Vera in a Patient With Heterozygous Beta-Thalassemia: Coincidence or Causal Relationship?

**DOI:** 10.7759/cureus.11589

**Published:** 2020-11-20

**Authors:** Konstantinos Kottas, Anastasios Marathonitis, Aikaterini Nodarou, Georgios Kanellis, Konstantinos Christopoulos

**Affiliations:** 1 Internal Medicine, Sismanogleio Hospital, Athens, GRC; 2 Hematopathology, Evangelismos General Hospital, Athens, GRC

**Keywords:** β-thalassemia, masked polycythemia vera, polycythemia vera, heterozygous beta thalassemia, pv + hbt, stress erythropoiesis, eryhropoiesis, pv, hbt

## Abstract

Polycythemia vera (PV) and heterozygous beta-thalassemia (HBT) have opposing effects on the hematocrit (Hct) and may mask the presence of each other. Missing the diagnosis of PV may have serious consequences, mainly by exposing the patient to the risk of thromboses. We present a case where the diagnosis of PV was delayed due to the coexistence of HBT, and review the relevant literature. It can be postulated that “stress erythropoiesis”, known to occur in patients with thalassemic syndromes, increases the probability of somatic JAK2 mutations leading to development of PV.

## Introduction

The thalassemic syndromes are monogenic disorders prevalent in many parts of the world and characterized by defective synthesis of one or more globin chains of the hemoglobin (Hb) molecule. Beta-thalassemia is caused by mutations in the beta-globin gene and is particularly common in the Mediterranean, the Middle East, and North Africa. The homozygous state (beta-thalassemia major) is characterized by complete absence of beta-globins, leading to severe, transfusion-dependent anemia. Thalassemia intermedia is a clinical classification which includes all the heterogeneous phenotypes of decreased beta-globin production which, occasionally, may lead to transfusion need. The heterozygous state (heterozygous beta-thalassemia, HBT), also known as beta-thalassemia trait or beta-thalassemia minor usually manifests as mild, asymptomatic anemia not requiring any specific treatment. The complete blood count in HBT typically shows microcytosis (resulting from α/β-chain imbalance) combined with erythrocytosis (reflecting chronic ineffective erythropoiesis). The diagnosis of HBT is confirmed by finding an increased percentage of Hb A2 on hemoglobin (Hb) electrophoresis or column chromatography. Although microcytosis is also seen in iron deficiency anemia, erythrocytosis is not a feature of the latter. Practically, in a patient with microcytic erythrocytosis, the only differential diagnoses to consider are other hemoglobinopathies (mainly alpha-thalassemia trait) and polycythemia coexisting with iron deficiency.

Polycythemia vera is a myeloproliferative neoplasm characterized by erythrocytosis driven by constitutive activation of the erythropoietin (Epo) receptor by mutant JAK2 protein kinase. It is associated with an increased risk of arterial and venous thromboses, especially in older patients and those with coexistent cardiovascular risk factors. Iron deficiency is found in the majority of PV cases at diagnosis or at some point during the course of the disease [1]. Causes include excessive iron utilization by the hyperplastic erythropoiesis, gastrointestinal blood loss, and therapeutic venesection.

We herein report a case in which the presence of HBT led to delayed diagnosis of PV in an elderly patient at high risk for thrombosis. We review the literature regarding the coexistence of HBT and PV, and discuss the hypothesis of a causal association between the two conditions.

## Case presentation

An 84-year-old woman presented with a four-week history of dizziness, headaches, and vertigo. Her past medical history included hypercholesterolemia, hypothyroidism, depression and osteopenia, and her regular medication consisted of atorvastatin, levothyroxine, citalopram, calcium, and cholecalciferol. She was a nonsmoker and had no history of cardiac, pulmonary, or renal disease. There was a family history of HBT but no other hematological disorders. The patient was known to have HBT with steady Hb and hematocrit (Hct) values marginally below the lower limit of normal, on yearly check-ups until two years prior to her presentation. A routine complete blood count arranged by her primary care physician nine months prior to presentation had shown Hb 14 g/dL, Hct 45.6%, red cell count (RBC) 6.13 x 1012/L with microcytosis and normal white cell and platelet counts. 

Physical examination revealed no remarkable findings. Her arterial oxygen saturation was 97%. Abdominal ultrasound examination showed a fatty liver and normal-sized spleen. Computerized brain tomography revealed changes of ischemic leukoencephalopathy. Blood tests showed RBC 7.01 x 1012/L, Hb 15.8 g/dL, Hct 50.4%, mean corpuscular volume (MCV) 69 fL, white cell count 6.76 x 109/L, and platelet count 236 x 109/L. Serum lactate dehydrogenase was 326 U/L (normal range 135-225 U/L), while the rest of her biochemistry profile was unremarkable. Serum levels of iron, ferritin, and vitamin B12 were normal. Hb electrophoresis showed an elevated HbA2 of 3.8% (reference range 1.8-3.2), fetal hemoglobin (HbF) of 0.5% (reference <2%) and no evidence of abnormal Hbs, which was compatible with HBT. Serum Epo levels were low at 3.0 mU/mL (reference range 4.3-29.0 mU/mL). Bone marrow biopsy appearances were compatible with a myeloproliferative neoplasm of the PV type, without excess fibrosis or increased numbers of immature cells. The diagnosis of PV was confirmed by identifying heterozygocity for the JAK2 V617F mutation in peripheral blood white cells, using a real-time polymerase chain reaction assay with hybridization probes and melting curve analysis.

The patient showed a good clinical and laboratory response to the administration of low-dose aspirin and hydroxyurea with a target Hct of 42%.

## Discussion

The present case demonstrates that the Hb and Hct values included in the major diagnostic criteria for PV according to the 2016 revised World Health Organization guidelines (Hb >16.5 g/dL in males and >16 g/dL in females, or Hct >49% in males and >48% in females) cannot be relied upon for timely diagnosis of PV in the presence of HBT [2]. In these patients, elevations of Hb or Hct above their steady state values should prompt a search for other diagnostic criteria, including suppressed serum Epo levels, presence of the JAK2 V617F mutation, and trilineage bone marrow hyperplasia.

There have been five previous reports of PV in patients with HBT (Table 1) [3-7]. So far, there is no epidemiological evidence indicating an association between PV and HBT. However, the latter is associated with chronic stimulation of erythropoiesis, as evidenced by increased reticulocyte counts and serum Epo levels. Paterakis et al. showed that, compared with normal controls, subjects with HBT had significantly higher reticulocyte counts, which were positively correlated with the degree of anemia [8]. This is in agreement with the findings of Vedovato et al., who found that levels of serum Epo were higher in HBT subjects, compared to those of normal controls [9]. It can be postulated that higher levels of Epo result in increased stimulation of the EpoR/JAK2/STAT5 signaling pathway, thus increasing the probability of JAK2 mutations occurring as a result of increased transcriptional activity of the JAK2 gene.

**Table 1:** Clinical and laboratory data of reported cases of coexistent HBT and PV. *The patient was heterozygous for beta-thalassemia and sickle cell disease. RBC, red blood cell; Hb, hemoglobin; Hct, hematocrit; MCV, mean corpuscular volume; Epo, erythropoietin; NA, not available; ND, not done; HBT, heterozygous beta-thalassemia; PV, polycythemia vera

	Present case	De Sloovere et al. (2015) [7]	Lopes da Silva and Silva (2011) [6]	Ifran et al. (2007) [5]	Thomas (2001) [4]	Castro (1981) [3]
Age	84	60	75	68	71	48
Gender	Female	Female	Female	Female	Female	Female
Hyperviscosity symptoms	Yes	Yes	No	Yes	Yes	No
Splenomegaly	No	Yes	Yes	Yes	No	Yes
RBC (x10^12^/L)	7.0	10.5	9.0	9.3	7.4	8.0
Hb (g/dL)	15.8	19.8	15.4	15.5	15.6	14.4
Hct (%)	50.4	58.6	59.2	59.0	48.1	42.0
MCV (fl)	69	56	59	NA	65	70
HbA2 (%)	3.8	4.1	4.3	4.7	5.5	4.9*
Epo	Low	Low	Low	Low	Low	ND
JAK2 V617F	Yes	Yes	Yes	ND	ND	ND

There are no published data regarding the prevalence of JAK2 mutations in HBT. Studies in small cohorts of patients with other thalassemic syndromes have given conflicting results: Taher et al. did not find the JAK2 V617F mutation in any of 36 Lebanese patients with thalassemia intermedia [10]. Similarly, Vlachaki et al. detected no JAK2 V617 mutation in 20 Greek beta-thalassemia patients with thrombocytosis following splenectomy [11]. At variance with these negative results, Asadi et al. have recently detected this mutation in 19% of 75 patients with beta-thalassemia major [12].

The fact that all reported HBT patients who developed PV were female (Table 1) has a small probability (p<0.05) of being coincidental. One is therefore tempted to speculate that women with HBT are more susceptible to the emergence of JAK2 mutations, compared with their male counterparts. Interestingly, this hypothesis is supported by the findings of Vedovato et al., who observed that women with HBT had significantly higher serum Epo levels compared to men with HBT [9]. All of the above indicate that a causal relationship between HBT and PV is plausible.

## Conclusions

Polycythemia vera coexisting with HBT may elude diagnosis for a considerable time, exposing the patient to the risk of serious complications. Timely diagnosis of PV in this population requires a high index of suspicion and a low threshold for investigation, including search for JAK2 mutations. The prevalence of the latter in individuals with HBT, and, vice versa, the prevalence of HBT in patients with myeloproliferative neoplasms bearing JAK2 mutations should be the subject of future research.
